# Working conditions mediate the association between social class and physical function in older age in Sweden: a prospective cohort study

**DOI:** 10.1186/s12889-020-09431-9

**Published:** 2020-09-04

**Authors:** Nikita Pandey, Alexander Darin-Mattsson, Charlotta Nilsen

**Affiliations:** 1 Aging Research Center (ARC), Karolinska Institutet/Stockholm University, SE-171 65 Stockholm, Sweden; 2 Stress Research Institute, Stockholm University, SE-106 91 Stockholm, Sweden

**Keywords:** Psychosocial working conditions, Physical working conditions, Older age, Physical impairment, Mobility, Physical function, Social class, Healthy aging, Longitudinal, Sweden

## Abstract

**Background:**

Global demographics are changing as societies all over the world are aging. This puts focus on maintaining functional ability and independence into older age. Individuals from lower social classes are at greater risk of developing limitations in physical function later in life. In this study, we investigated the mediating role of working conditions in the association between occupation-based social class and physical function measured as self-reported mobility limitations and objectively measured physical impairment in older age.

**Methods:**

Two Swedish surveys, linked at the individual level, were used (*n* = 676–814 depending on the outcome). Follow-up time was 20–24 years. Multiple logistic regression analyses were performed with adjustments for age, sex, level of education, mobility, and health problems at baseline. This was followed by analyses of the size of the mediating effect of working conditions.

**Results:**

Working conditions seem to mediate 35–74% of the association between social class and physical impairment in older age. The pattern of mediation was primarily driven by passive jobs, i.e., low psychological demands and low control, among blue-collar workers. Working conditions did not mediate the association between social class and self-reported mobility limitations in older age.

**Conclusions:**

The results of this study indicate that working conditions are important in combating the social gradient in healthy aging, contributing to the evidence regarding the magnitude of impact exerted by both the physical and psychosocial work environment separately and in conjunction.

## Background

Global demographics are changing as societies are aging. Within the EU, it is estimated that nearly 30% of the total population will be 65 years or older by 2060 [1]. While this is a good indication of the success of modern medicine, it raises major concerns regarding global preparedness to accommodate the ensuing shift in social dynamics. Recognizing the increasing care need that follow a growing older population [2], a global strategy and action plan for aging and health was published [1], defining healthy aging as “the process of developing and maintaining the functional ability that enables wellbeing in older age”. Evidence suggests that limited physical function restricts such active participation of older adults within society [3]. Physical function decreases with age. Particularly in the age group of 80+ [4]. Deterioration of physical function, such as muscle function, aerobic capacity, and postural balance [5] leads to impaired ability to perform daily activities [6] and has been associated with reduced quality of life [7]. Therefore, to preserve physical function in older adults is a major public health concern.

A staggering amount of research confirms that individuals from lower social classes are at greater risk of developing adverse health outcomes in older age than those from higher social classes [8–10]. Health inequality persists across populations throughout the world. Variations in physical function in older age follow a similar pattern [11]. As suggested by the accumulation of advantage and disadvantage (CAD) theory [12], the chain of disadvantages derived from belonging to a lower social class throughout the life course have been shown to further induce health inequalities in later life [10]. For example, working conditions are often a consequence of an individual’s occupational social class. People who belong to a lower social class, such as blue-collar workers, are more likely to be exposed to adverse psychosocial working conditions throughout work life, including work-related stress, repetitive work, low influence at work, as well as physically demanding working conditions [13]. Hence, work is an area that is central to reducing inequalities in health outcomes [14]. There is also growing evidence of a long-term association between adverse working conditions and limitations in physical function in older age that persist after adjusting for socioeconomic factors [15–17]. Chronic stress derived from the work environment may cause damage to the physical body via disruptions of metabolism, blood pressure, hormone, and immune function [18] that may, in turn, negatively impact physical function in older age. Moreover, exposure to high physical workload has been associated with musculoskeletal pain and lower physical function in retirees that may cause long-term physical damage to the body [19].

The role of working conditions on the social gradient of health is well-established [20]; however, the objectives, parameters, and methodologies used in evaluating this relationship largely differ [21–23]. Among studies that examine the mediating role of working conditions in relation to social class, a common drawback is the lack of long-term evidence, as most studies either have a short period of follow-up or measure the outcomes among cohorts that retain generally good physical function by excluding the frail oldest old. Our study aims to overcome these challenges by utilizing a long period of follow-up and measuring the outcomes when the participants have reached a high age and a high prevalence of physical limitations. In this study, the role of working conditions on the social gradient of physical function in older age was assessed 20 years later. The prospective nature of the data used, and the high participant response rates, provide a unique opportunity to examine long-term associations. For this purpose, the following research questions were investigated: Is social class associated with late-life physical function 20–24 years later? If so, do physical and psychosocial working conditions mediate such association?

## Methods

### Data and analytic sample

Two linked nationally representative surveys conducted in Sweden were used: The Level of Living Survey (LNU) [24] and the Swedish Panel Study of Living Conditions of the Oldest Old (SWEOLD) [25]. The first LNU sample was selected in 1968. New waves were conducted in 1974, 1981, 1991, 2000 and 2010. The age range of the sample was 18–75 years. To represent the Swedish population, randomized quota sampling was conducted to include immigrants and young people. When crossing the upper age limit of 75 years, participants were re-interviewed in the SWEOLD study, which was conducted in five waves: 1992, 2002, 2004, 2011 and 2014. For LNU, structured face-to-face interviews were conducted with professional interviewers. SWEOLD primarily followed the same procedure. However, if a participant had a cognitive impairment or was unable to participate, proxy interviews were conducted with either the spouse, close relative, friend or relevant healthcare professional.

Data used in this study comprises three linked sets of data combined into one longitudinal dataset. Data from LNU 1968 were linked with re-interviews of the sample in SWEOLD 1992 (linkage 1), data from LNU 1981 were linked with SWEOLD 2002 (linkage 2) and data from LNU 1991 were linked to SWEOLD 2011 (linkage 3). The three linkages were compiled in one dataset and analyzed together. If respondents from the baseline in 1968 or 1981 did not respond to any of the questions, the same respondent’s answer from LNU 1974 was used instead. Non-response in baseline 1991 was replaced with answers from LNU 1981. However, this imputation did not exceed 10% in any variable. Response rates in LNU range from 78 to 91%, and in SWEOLD, from 86 to 95%.

**Fig. 1:**
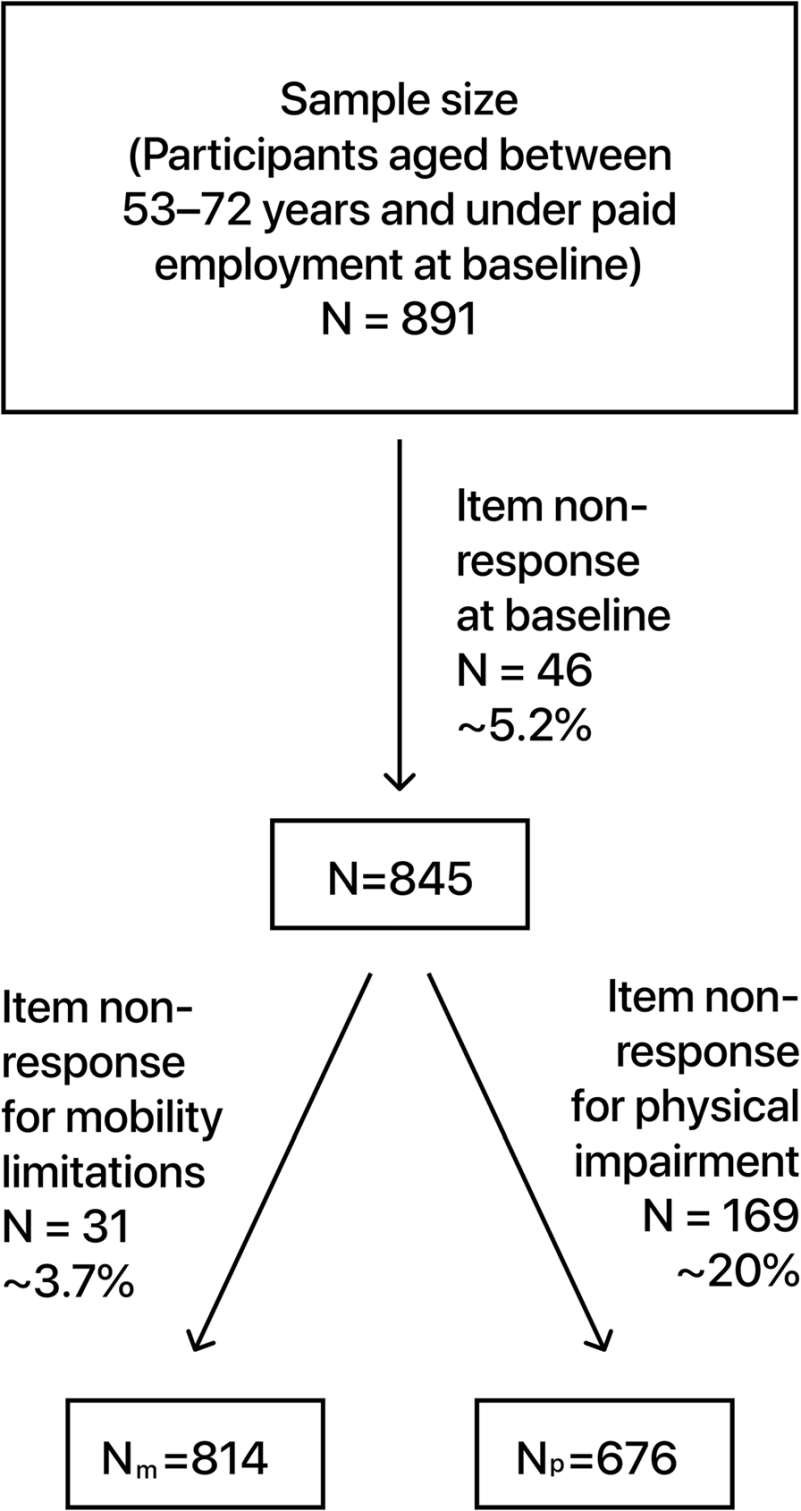
Sample size flowchart

In order to follow-up the participants from LNU in SWEOLD, they had to be 53 years or older at baseline. Long-term unemployed, students and housewives were excluded from the analyses because their working conditions could not be assessed. Baseline item nonresponse (*n* = 46, 4.9%) reduced the sample from 891 to 845 participants and item nonresponse at follow-up further reduced it to 676 or 814 depending on outcomes (Fig. 1). Age varied between 53 and 72 years (mean age 59) at baseline and 77 and 95 years (mean age 81) at follow-up.

### Measures of physical function in older age

The main outcome variable was physical function in older age measured using two components available in the SWEOLD study: mobility limitations and physical impairment, used in earlier research [8, 15–17, 19]. *Mobility limitations* were assessed with the responses to three dichotomous “yes/no” questions: “Can you walk 100 meters briskly without difficulty?”, “Can you walk up and down stairs with no problem?”, and “Can you stand without support?”. The combined responses were coded into a three-value ordinal scale wherein 0 = “no mobility limitations” (completed all activities without difficulty), 1 = “mild” (unable to complete one of the activities without difficulty), and 2 = “severe” (difficulty in completing two or all three activities). *Physical impairment* was assessed by combining nine objective tests as follows: Picking up a pen from the floor, lifting 1 kilogram, touching the left ear with the right hand, touching the right ear with the left hand, touching the left toes with the right hand, touching the right toes with the left hand, placing both hands under the thighs/bottom, turning both palms up and down and getting up from a chair with arms crossed. The respondents were categorized on each task as “managed without difficulty,” “managed with difficulty,” and “did not manage.” To increase power, the resultant variable was dichotomized (“absent” i.e. participant managed all tasks without help, and “present” participant was unable to manage all tasks without help. The participants’ responses on Activities of Daily Living (ADL), recorded in the SWEOLD surveys, were used to impute missing values. Participants who were unable to carry out one or more ADL (e.g. dressing/undressing, bathroom visits, eating) were coded as “did not manage” based on the theoretical assumption that they were unable to participate in the physical performance tests (*n* = 34).

### Measures of social class

*Occupational social class* was assessed at baseline. Occupation was categorized in accordance with the official Swedish socioeconomic classification (SEI) made by Statistics Sweden, and have been previously published [8, 10, 16]. This classification resembles the internationally well-known Erikson, Goldthorpe, and Portocarero’s (EGP) schema [26]. In order to include small-scale farmers and entrepreneurs, as well as allocate ranks and make social class an ordinal variable, the SEI categories for self-employed individuals were regrouped. The social class variable was coded as unskilled blue-collar workers, skilled blue-collar workers, including small-scale farmers and entrepreneurs with no employees, lower white-collar workers, including medium-scale farmers and entrepreneurs with one to nine employees, and upper white-collar workers, including large-scale farmers, academic professionals and entrepreneurs with at least 10 employees. The SEI coding incorporates educational achievement in the ordering of occupations as part of the classification criteria.

### Measures of working conditions

*Physical working conditions* were assessed at baseline using a six-item index consisting of exposure to various components during the week before the interview. These were: “Do you have to be capable of lifting 60 kilos (heavy lifting) at your work?” (yes/no), “Are you exposed to heavy vibrations at your work?” (yes/no), “Is your job physically demanding?” (yes/no), “Do you sweat every day?” (yes/no), “Are you exposed to poison/acid/explosives?” (yes/no), “Are you exposed to gas, smoke or dust at your work?” (yes/no). The responses were then grouped into three categories. If participants responded negatively to all questions, they were coded as “no exposure”; if they responded “yes” to two or less questions, they were coded as “low exposure” and positive responses to more than two questions were coded as “high exposure.” This categorization was made to preserve power as few participants were exposed to all components. *Psychosocial working conditions* were assessed at baseline through responses to self-reported yes/no questions and categorized based on the job demand-control model [27]. Demand was derived from the questions “Is your job psychologically demanding/taxing?” and “Is your job hectic?” (yes/no). To answer “yes” to both questions was classified as psychologically demanding. Control consists of personal schedule freedom (decision-making authority) and intellectual discretion (skill discretion). In this study, control was measured through intellectual discretion exclusively, by the questions “What is the level of education required by your job?” and “Is your job monotonous?”. Based on the combined responses, jobs were categorized as: repetitious/monotonous, not repetitious/monotonous and minimum skill level, not repetitious/monotonous and 1–4 years of training (skill level required); and not repetitious/monotonous and more than 4 years of training (skill level required). This was dichotomized into low and high control, in which repetitious/monotonous work and non-repetitious/monotonous work with a minimum skill level was coded as “low control” and the other two were coded as high control.” The demand and control variables were then combined to form four job categories: “passive” = low control and low demand, “high-strain” = low control and high demand, “low-strain” = high control and low demand, and “active” = high control and high demand.

### Covariates

*Age* (continuous) and sex (binary) were retrieved from the registers and confirmed during the interviews. *Mobility at baseline* was created in the same manner as described for mobility limitations in old age. *Health problems at baseline* were assessed using an index of self-reported diseases and symptoms experienced over the last 12 months (pain in shoulders, back, hips, joints and/or stomach, cardio-vascular diseases and symptoms, including hypertension, chest pain, swollen legs, myocardial infarction and heart failure, diabetes, leg ulcer, dizziness, breathlessness, fatigue and sleep problems). *Education* was assessed by highest level of education at baseline and divided into two groups: compulsory and beyond compulsory (i.e., vocational, upper secondary, and university).

### Statistical analyses

Stata version 14 was used for all analyses. Binary and ordered logistic regression were used to investigate associations between independent and dependent variables. The proportional odds assumption was tested using generalized ordered logistic regression and approved. To test if the three linkages could be merged into one, we analyzed the linkages separately. Although estimate size and significance level slightly varied between the three linkages, the associations were in the same direction. Also, interaction between linkage and social class was tested. The interactions were statistically non-significant. Thus, the three linkages were merged into one data set to increase power. Supplementary analyses were conducted by including an interaction term between sex and social class. The interaction term was only statistically significant for one out of the 28 interactions, likely by chance. The only statistically significant interaction showed that women in lower white-collar occupations reported more mobility limitations in older age than men. Thus, we analyzed women and men together. All analyses were adjusted for baseline characteristics: age, sex, mobility and health problems (Model 1). Model 2 was additionally adjusted for physical working conditions. Model 3 was adjusted like Model 1 and additionally for psychosocial working conditions. Model 4 was adjusted like model 1 and additionally for physical and psychosocial working conditions.

KHB (Karlson, Holm, and Breen) is a user command in STATA that conducts mediation analyses of a variable in the relationship between an independent and dependent variable by comparing the beta coefficients of two nested non-linear probability models [28]. The output is in the form of total effects, direct effects and indirect effects. When there is an indirect effect, but no direct effect, it is called ‘full mediation’. When there are both direct and indirect effects, it is called ‘partial mediation’ [29].

## Results

### Population characteristics

Table 1 illustrates that blue-collar workers had the greatest exposure to adverse physical working conditions. Moreover, almost one half of the analytic sample (46.6%) was employed in passive jobs and unskilled blue-collar workers had the highest percentage (71.5%). Around one-fifth of unskilled blue-collar workers (21.4%) suffered from severe health problems at baseline and this proportion decreased in magnitude with every consecutive social class to 6.8% among upper white-collar workers. A similar pattern was observed with mobility limitations measured at baseline. About 82% among upper white-collar workers had an education level beyond compulsory, while the frequency was only 22% among unskilled blue-collar workers.

**Table 1:** Descriptive statistics at baseline

Occupational social class					
	Unskilled blue-collar	Skilled blue-collar	Lower white-collar	Upper white-collar	Total (n)
**Total,** n (%)^a^	285 (33.73)	158 (18.70)	197 (23.31)	205 (24.26)	845
**Mean age** (SD)	59 (3.30)	60 (3.87)	60 (3.38)	59 (3.22)	59 (3.42)
**Sex,** n (%)^b^					
Women	190 (66.7)	35 (22.2)	96 (48.7)	74 (36.1)	395 (46.7)
Men	95 (33.3)	123 (77.8)	101 (51.3)	131 (63.9)	450 (53.3)
**Physical working conditions,** n (%)^b^					
No exposure	63 (22.1)	31 (19.6)	100 (50.8)	137 (66.8)	331 (39.2)
Low exposure	147 (51.6)	52 (32.9)	51 (25.9)	58 (28.3)	308 (36.4)
High Exposure	75 (26.3)	75 (47.5)	46 (23.3)	10 (4.9)	206 (24.4)
**Psychosocial working conditions,** n (%)^b^					
Passive	204 (71.5)	94 (59.5)	78 (39.6)	18 (8.8)	394 (46.6)
High-strain	43 (15.1)	18 (11.4)	39 (19.8)	19 (9.2)	119 (14.1)
Low-strain	21 (7.4)	34 (21.5)	50 (25.4)	83 (40.5)	188 (22.2)
Active	17 (6.0)	12 (7.6)	30 (15.2)	85 (41.5)	144 (17.0)
**Level of education**, n (%)					
Compulsory	160 (78.1)	97 (49.2)	48 (30.4)	51 (17.9)	356 (42.1)
Beyond compulsory	45 (22.0)	100 (50.8)	110 (69.6)	234 (82.1)	489 (57.9)
**Health problems at baseline,** n (%)^b^					
None or mild	224 (78.6)	134 (84.8)	173 (87.8)	191 (93.2)	722 (85.4)
Severe	61 (21.4)	24 (15.2)	24 (12.2)	14 (6.8)	123 (14.6)
**Mobility at baseline,** n (%)^b^					
Unrestricted	214 (75.1)	126 (79.7)	159 (80.7)	183 (89.3)	682 (80.7)
Restricted	71 (24.9)	32 (20.3)	38 (19.3)	22 (10.7)	163 (19.3)

*SD* Standard deviation, *n* Analytic size

^a^Row percentage

^b^Column percentage

Table 2 illustrates a clear gradient in mobility limitations in older age, where unskilled blue-collar workers reported the most severe mobility limitations (28.2%) and upper white-collar workers reported the least (12.0%). The proportion between those who managed performance tests and those who did not differed between unskilled blue-collar workers (49.3%) and upper white-collar workers (27.9%).

**Table 2:** Descriptive statistics at follow-up

	Occupational social class									
	Unskilled blue-collar		Skilled blue-collar		Lower white-collar		Upper white-collar		Total n (%)	
Age (years)										
Mean (SD)	81 (3.20)		81 (3.72)		80 (3.31)		80 (3.05)		81 (3.31)	
Sex, n (%)										
Women	190 (66.7)		35 (22.2)		96 (48.7)		74 (36.1)		395 (46.7)	
Men	95 (33.3)		123 (77.8)		101 (51.3)		131 (63.9)		450 (53.3)	
Mobility limitations, n (%)										
None	135	(49.5)	80	(52.3)	105	(55.8)	134	(67.0)	454	(55.8)
Mild	61	(22.3)	33	(21.6)	38	(20.2)	42	(21.0)	174	(21.4)
Severe	77	(28.2)	40	(26.1)	45	(24.0)	24	(12.0)	186	(22.8)
Physical Impairment, n (%)										
Absent	110	(50.7)	68	(55.7)	86	(54.4)	129	(72.1)	393	(58.1)
Present	107	(49.3)	54	(44.3)	72	(45.6)	50	(27.9)	283	(41.9)

*SD* Standard Deviation, *n* Analytic size

### Social class and physical function in older age

Although all associations did not meet traditional thresholds for declaring statistical significance (*p* < .05), the basic pattern emerging in Table 3 suggest that in reference to upper white-collar workers, all other social classes were associated with greater odds of developing limited physical function in older age (i.e. self-reported mobility limitations and objective tests of physical impairment) when adjusted for covariates. Particularly, skilled blue-collar workers had higher odds of developing mobility limitations (OR 1.73, CI 1.09–2.73) and lower white-collar workers had higher odds of developing physical impairment (OR 1.67, CI 1.04–2.68) in older age (Table 3, model 1). On further adjusting for physical working conditions at baseline (model 2), the association between occupational social class and mobility limitations strengthened, but showed attenuation with physical impairment (Table 3, model 2).

**Table 3:** Association between occupational social class and physical function in older age

	Physical function in older age			
	Mobility limitations		Physical impairment	
	OR	95% CI	OR	95% CI
**Model 1**				
Upper white-collar^a^ ref.		ref.	ref.	ref.
Lower white-collar	1.34	0.88–2.05	1.67^*^	1.04–2.68
Skilled blue-collar	1.73^*^	1.09–2.73	1.65^†^	0.97–2.79
Unskilled blue-collar	1.51^†^	0.98–2.31	1.52^†^	0.93–2.48
**Model 2 Physical working conditions**				
Lower white-collar	1.45^†^	0.94–2.22	1.55^†^	0.95–2.52
Skilled blue-collar	2.01^**^	1.23–3.26	1.43	0.81–2.50
Unskilled blue-collar	1.74^*^	1.10–2.75	1.33	0.79–2.23
**Model 3 Psychosocial working conditions**				
*Model 3a Passive*				
Lower white-collar	1.36	0.88–2.08	1.52^†^	0.94–2.48
Skilled blue-collar	1.75^*^	1.09–2.82	1.43	0.82–2.48
Unskilled blue-collar	1.53^†^	0.97–2.41	1.25	0.74–2.13
*Model 3b High-strain*				
Lower white-collar	1.29	0.85–1.97	1.68^*^	1.04–2.71
Skilled blue-collar	1.75^*^	1.11–2.77	1.64^†^	0.97–2.79
Unskilled blue-collar	1.47^†^	0.96–2.26	1.52^†^	0.93–2.49
*Model 3c Low-strain*				
Lower white-collar	1.28	0.84–1.96	1.60^†^	0.99–2.58
Skilled blue-collar	1.67^*^	1.05–2.65	1.56^†^	0.93–2.70
Unskilled blue-collar	1.37	0.89–2.12	1.38	0.83–2.27
*Model 3d Active*				
Lower white-collar	1.37	0.89–2.11	1.68^*^	1.03–2.73
Skilled blue-collar	1.77^*^	1.11–2.84	1.66^†^	0.96–2.86
Unskilled blue-collar	1.55^†^	0.99–2.41	1.54^†^	0.92–2.55
**Model 4 Physical and psychosocial working conditions**				
Lower white-collar	1.36	0.87–2.12	1.42	0.86–2.37
Skilled blue-collar	2.01^**^	1.21–3.35	1.28	0.71–2.31
Unskilled blue-collar	1.61^†^	0.98–2.63	1.10	0.62–1.96

*p* < 0.10^†^, *p* < 0.05*, *p* < 0.01**. *OR* Odds Ratio, *CI* Confidence Interval. All models are adjusted for age, sex, level of education, mobility, and health problems at baseline (Model 1). Model 2 was additionally adjusted for physical working conditions. Model 3 was adjusted like Model 1 and additionally for psychosocial working conditions. Model 4 was adjusted like Model 1 and additionally for physical and psychosocial working conditions. ^a^The reference category is upper white-collar workers in all models

Adjusting for psychosocial working conditions did not alter the association between social class and mobility limitations in older age (Table 3, model 3b, and 3d). However, adjusting for passive or low-strain jobs attenuated the association between social class and physical impairment in older age (Table 3, model 3a and 3c).

After adjusting for both physical and psychosocial working conditions in model 4 (Table 3), the association between social class and physical impairment in older age became statistically non-significant.

#### The mediating role of working conditions

Decomposing the association between social class and physical function in older age (i.e. mobility limitations and physical impairment) using physical and psychosocial working conditions assessed separately revealed that the indirect effects did not reach traditional threshold for statistical significance (*p* < .05) (Table 4). However, a negative mediation effect was observed among blue-collar workers (*p* < .10) in relation to mobility limitations in older age. Among blue-collar workers, − 26.4% (skilled blue-collar workers) and − 31.9% (unskilled blue-collar workers) of the total effect seem to be attributable to physical working conditions (“partial mediation,” i.e. both direct and indirect effects). Moreover, passive jobs mediated (*p* < .10) the association between blue-collar workers and physical impairment in older age (Table 4, model 3a). Among blue-collar workers, 27.6% (skilled blue-collar workers) and 45.7% (unskilled blue-collar workers) of the total effect is attributable to passive jobs (“full mediation,” i.e. indirect effect only; Table 4, model 3a). Low-strain jobs mediated (*p* < .10) the association between unskilled blue-collar workers and mobility limitations by 23.38% (full mediation; Table 4, model 3c). High-strain jobs or active jobs did not mediate the association between social class and physical impairment in older age (Table 4, model 3b, and 3d).

**Table 4:** Decomposition of effects of social class on physical function in older age

	Mediation analysis of working conditions							
	Mobility limitations				Physical impairment			
	Total effects	Direct effects	Indirect effects	Mediation %^a^	Total effects	Direct effects	Indirect effects	Mediation %^a^
**Model 2 Physical working conditions** *(ref: upper white-collar)*								
Lower white-collar	0.30	0.37^†^	−0.07	−23.92	0.51^*^	0.44^†^	0.08	14.63
Skilled blue-collar	0.55^*^	0.70^**^	−0.15^†^	− 26.40	0.50^†^	0.36	0.14	28.80
Unskilled blue-collar	0.42^†^	0.55^*^	− 0.14^†^	−31.89	0.42^†^	0.28	0.14	32.50
**Model 3 Psychosocial working conditions**								
*3a Passive (ref: upper white-collar)*								
Lower white-collar	0.30	0.30	0.00	−3.05	0.51^*^	0.42^†^	0.09	17.59
Skilled blue-collar	0.55^*^	0.56^*^	−0.01	−2.50	0.49^†^	0.36	0.14^†^	27.61
Unskilled blue-collar	0.41^†^	0.43^†^	−0.02	−4.32	0.42^†^	0.23	0.19^†^	45.71
*3b High-strain (ref: upper white-collar)*								
Lower white-collar	0.30	0.26	0.04	14.41	0.51^*^	0.52^*^	−0.01	−1.64
Skilled blue-collar	0.56^*^	0.56^*^	−0.00	−0.11	0.50^†^	0.50^†^	0.00	0.01
Unskilled blue-collar	0.41^†^	0.39^†^	0.02	5.98	0.42^†^	0.42^†^	−0.00	−0.91
*3c Low-strain (ref: upper white-collar)*								
Lower white-collar	0.29	0.25	0.04	15.20	0.51^*^	0.47^†^	0.04	8.50
Skilled blue-collar	0.55^*^	0.51^*^	0.04	6.54	0.50^†^	0.46^†^	0.04	7.19
Unskilled blue-collar	0.41^†^	0.37	0.10^†^	23.38	0.42^†^	0.32	0.10	23.92
*3d Active (ref: upper white-collar)*								
Lower white-collar	0.30	0.32	−0.02	−8.13	0.51^*^	0.52^*^	−0.01	−1.61
Skilled blue-collar	0.54^*^	0.57^*^	−0.03	−5.15	0.50^†^	0.51^†^	−0.01	−1.97
Unskilled blue-collar	0.41^†^	0.53^†^	−0.03	−7.33	0.42^†^	0.43^†^	−0.01	−2.57
**Model 4 Physical and psychosocial working conditions** *(ref: upper white-collar)*								
Lower white-collar	0.31	0.28	0.02	7.92	0.52^*^	0.33	0.18^†^	35.35
Skilled blue-collar	0.56^*^	0.60^*^	−0.04	−8.00	0.50^†^	0.25	0.26^*^	50.91
Unskilled blue-collar	0.43^†^	0.42^†^	0.01	3.00	0.42^†^	0.11	0.31^*^	73.69

^a^Mediation % corresponds to conf_pct in khb decomposition output in STATA 14 which stands for confounding percentage net of rescaling *p* < 0.10^†^, *p* < 0.05*, *p* < 0.01**. Effect size expressed as beta coefficients unless otherwise specified. All models were adjusted for age, sex, level of education, mobility, and health problems at baseline (Model 1). Model 2 was additionally adjusted for physical working conditions. Model 3 was adjusted like Model 1 and additionally for psychosocial working conditions. Model 4 was adjusted like Model 1 and additionally for physical and psychosocial working conditions

An investigation of the combined mediating role of physical and psychosocial working conditions between social class and physical impairment in older age showed full mediation, in which both physical and psychosocial working conditions contributed. With upper white-collar workers as a reference, 35% of the total effect was attributable to working conditions among lower white-collar workers (*p* = .051; 13% attributable to physical working conditions and 23% attributable to psychosocial working conditions). Although the total effect did not reach traditional thresholds for statistical significance (*p* < .05), the indirect effect suggest that 51% of the total effect among skilled blue-collar workers was attributable to working conditions (25% attributable to physical working conditions and 26% attributable to psychosocial working conditions) and 74% of the total effect among unskilled blue-collar workers was attributable to working conditions (28% in physical working conditions and 45% in psychosocial working conditions; Table 4, model 4). No statistically significant mediating effects of working conditions were found in the association between social class and mobility limitations when physical and psychosocial working conditions were analyzed together (Table 4, model 4).

## Discussion

This study examined the association between occupational social class and physical function in older age and attempted to disentangle this association by quantifying the mediating role of working conditions. In short, upper white-collar workers had lower odds of physical impairment in older age when compared to lower social classes. Full mediation was observed by working conditions in the association between social class and physical impairment in older age, in which 35–74% of the total effect was attributable to working conditions. This mediating effect was primarily driven by passive jobs in blue-collar workers.

In accordance with previous research, our results show a social gradient in physical function in older age [30]. As expected, adverse physical working conditions are clustered in lower social groups, as are passive jobs (low demand combined with low control) [11]. The social gradient in health has previously been partially explained by several intermediary pathways such as material access, health behaviors, and working conditions [31, 32]. Our findings indicate that working conditions explain a high portion of the social gradient in physical impairment. This mediating effect was primarily driven by passive jobs in blue-collar workers. There is growing evidence of the long-term impact of adverse psychosocial working conditions on limitations in physical function in older age [15–17]. Passive jobs are unchallenging and may induce a loss of skills and unlearning. This could cause psychological atrophy and reduced self-efficacy. Low belief in own abilities has been linked to an unhealthy and passive lifestyle, e.g. passive jobs have been associated with a more passive lifestyle outside of work, such as physical inactivity [33]. Consequently, physical activity during leisure time may help preserve physical function in older age [34]. Moreover, passive jobs may be perceived as stressful. Understimulation has similar stress responses and symptoms to those that result from overstimulation [35]. Also, people in passive jobs can perceive even moderate psychological demands as stressful [36]. Increasing level of control at work, such as possibility to alter work positions/tasks (less monotonous work) and/or have the skills needed to be able to perform work tasks, may have beneficial effects on the physical body, as well as increasing level of self-efficacy. This, in turn, may also induce a more active lifestyle.

However, the weak support of physical working conditions as mediators was somewhat unexpected given previous research which suggested that physical working conditions play the largest role as mediator in the association between social class and physical function in older age [37, 38]. A potential explanation for our findings may be the inclusion of a more recent cohort, namely, those still employed in 1991. Improvements in the physical work environment in Sweden may have contributed to the drivers of physical function in older age being substituted by primarily psychosocial factors in the work environment. Post hoc analyses excluding the third linkage from the dataset confirmed this assumption. Although not reaching traditional thresholds for declaring statistical significance (*p* < .05), the patterns of the mediation analysis suggest that physical working conditions among blue-collar workers may be suppressing the true association between social class and mobility limitations in older age (*p* < .10). A suppressor has been defined as “a variable which increases the predictive validity of another variable (or set of variables) by its inclusion in a regression equation” [39]. This may be a consequence of the distribution of physical working conditions among social classes, with lower social groups characteristically exposed to a harsher physical work environment. Hence, failure to account for these working conditions may result in an underestimation of the true magnitude of the relationship between social class and self-reported mobility limitations in older age [40]. This interpretation could explain the role of physical working conditions in our regression models but as the indirect values were statistically non-significant (*p* < .05), it may be imprecise and inconclusive. Contrastingly, physical working conditions moderately attenuated the association between social class and physical impairment in older age. This may indicate that the advantage upper white-collar workers have over blue-collar workers, with respect to physical performance in older age, may be explained by the variations in physical environments across occupational classes. A possible explanation could be that the performance tests that evaluated physical performance were of the same body parts that undergo strain throughout working life in physically demanding jobs, compared to self-reported mobility limitations. Given that adverse physical working conditions are most common in blue-collar workers, as are passive jobs, the mediating effect of passive jobs may be influenced by observed and unobserved factors in the physical work environment. Variations in the role of the physical work environment on the social gradient of self-reported mobility limitations and objectively measured physical impairment highlight the need to capture both subjective and objective measures of outcome [41].

### Limitations and strengths

Strengths of this study include the high response rates in both the LNU (78–91%) and the SWEOLD (86–95%) survey, the use of a national random sample, the long follow-up (20–24 years) of the same participants and the inclusion of both self-reported mobility and objective tests of physical function as dependent variables [42, 43]. The data used have been extensively cited [8, 9, 44]. Nevertheless, the results should be interpreted with caution due to some methodological considerations. First, selective attrition must always be considered in longitudinal studies. The loss of participants due to mortality (selective survival, i.e. those who are better off survive longer) or dropout could lead to under- or overestimation of the true association. However, telephone and proxy interviews could help address the selective attrition bias by including the most disabled participants in the sample [45]. Also, imputations were performed for performance tests by using information gathered during interviews on ADL in order to minimize exclusion of older adults with the poorest health. Such imputations may introduce a misclassification bias. However, very few were imputed (5%) and it is very likely that a person that is not able to carry out one or more ADL would have severe difficulties to carry out the physical performance tests [6]. Another source of selection bias could be the ‘healthy worker effect’, i.e. people who have the capacity to work are retained in the workforce [46]. Including people that worked passed official retirement ages may have further strengthen this selection bias. This may result in an underestimation of the true association as people with worse health have left the workforce (possibly attributable to adverse working conditions) and have not been included in the study. Moreover, type of occupation may also be influenced by health status [47]. We did not have the possibility to adjust for health prior to baseline. However, adjusting for mobility and health problems at baseline is a way of accounting for at least some of these pre-existing conditions in order to minimize the effect of undetected reverse causation between health and occupation.

Second, the questions used for assessing psychosocial working conditions were limited. The original questionnaire was developed and expanded in 1991 to include other dimensions of work stress [48]. Unfortunately, the additional information needed for the expanded questionnaire was unavailable in the early waves of the Level of Living Surveys. As the cohorts of this study are from 1986 to 1991, they may not depict the most accurate image of current psychosocial work stress. However, these questions were used by Karasek to create the original demand-control model [27]. Recent research also indicates that the dimensions of demand and control continue to be a valid measure of the psychosocial work environment and are relevant to occupational health [49]. Finally, although the long gap between baseline and follow-up present some advantages with respect to the assessment of the long-term impact of social class on physical function, it also has limitations. Particularly as it does not reveal a possible change in social class or working conditions between baseline (mean age 59) and age at retirement. However, there was little work mobility in this cohort [50] and at the ages that working conditions were assessed [51], and most likely the participants have already reached their highest achieved occupation-based social class.

### Potential implications

The implications of this study extend to the working population with comparable social and political contexts. Globally, low skilled and non-routine manual labor corresponding to blue-collar jobs still make up 45% of total employment [52]. These groups will be major contributors to the aging population and we must find ways to encourage healthy aging among them. The early identification of harmful indicators in the work environment may help in the design of efficient workspaces for the future. Ongoing efforts towards creating safer physical work environments must be encouraged, but there is also a need to address the psychosocial aspects of the workplace.

## Conclusions

To conclude, the results highlight the importance of working conditions in combating the social gradient in physical function in older age, contributing to evidence on the magnitude of impact exerted by both the physical and psychosocial work environment separately and in conjunction. By following the same participants for over 20 years, this study reveals the need for a life-course perspective when investigating predictors of healthy aging.

## Data Availability

The datasets used and/or analyzed during the current study are available from the corresponding author on reasonable request. Public access to the database may be given if applicants have a scientific affiliation, sign a statement that the data will only be used for scientific purposes, and that the scientific project have ethical approval. Applicable sections of The Swedish Research Council (VR) principles for conducting research in humanities and the social sciences must be adhered to (http://www.codex.vr.se/en/forskninghumsam.shtml). Data will be available after the above-mentioned documents have been received, by e-mail at the SWEOLD Research Data Center dataaccess@sweold.se More information can be found at www.sweold.se.

## Abbreviations

ADL: Activities of Daily Living
CI: Confidence Interval
KHB: Karlson, Holm, and Breen command
LNU: The Level of Living Survey
OR: Odds Ratio
SEI: Swedish Socioeconomic Index
SWEOLD: The Swedish Panel Study of Living Conditions of the Oldest Old
